# Clinical Outcomes and Microbiological Characteristics of Severe Pneumonia in Cancer Patients: A Prospective Cohort Study

**DOI:** 10.1371/journal.pone.0120544

**Published:** 2015-03-24

**Authors:** Ligia S. C. F. Rabello, Jose R. L. Silva, Luciano C. P. Azevedo, Ivens Souza, Viviane B. L. Torres, Maíra M. Rosolem, Thiago Lisboa, Marcio Soares, Jorge I. F. Salluh

**Affiliations:** 1 Postgraduate Program of Internal Medicine – Universidade Federal do Rio de Janeiro, Rio de Janeiro, Brazil; 2 Intensive Care Unit, Hospital Sirio-Libanes, São Paulo, Brazil; 3 Intensive Care Unit, Hospital Barra Dor, Rio de Janeiro, Brazil; 4 Intensive Care Unit, Santa Casa de Misericórdia de Porto Alegre, Porto Alegre, Brazil; 5 D’Or Institute for Research and Education, Rio de Janeiro, Brazil; 6 Postgraduate Program, Instituto Nacional de Câncer, Rio de Janeiro, Brazil; University of Florida, UNITED STATES

## Abstract

**Introduction:**

Pneumonia is the most frequent type of infection in cancer patients and a frequent cause of ICU admission. The primary aims of this study were to describe the clinical and microbiological characteristics and outcomes in critically ill cancer patients with severe pneumonia.

**Methods:**

Prospective cohort study in 325 adult cancer patients admitted to three ICUs with severe pneumonia not acquired in the hospital setting. Demographic, clinical and microbiological data were collected.

**Results:**

There were 229 (71%) patients with solid tumors and 96 (29%) patients with hematological malignancies. 75% of all patients were in septic shock and 81% needed invasive mechanical ventilation. ICU and hospital mortality rates were 45.8% and 64.9%. Microbiological confirmation was present in 169 (52%) with a predominance of Gram negative bacteria [99 (58.6%)]. The most frequent pathogens were methicillin-sensitive *S*. *aureus* [42 (24.9%)], *P*. *aeruginosa* [41(24.3%)] and *S*. *pneumonia* [21 (12.4%)]. A relatively low incidence of MR [23 (13.6%)] was observed. Adequate antibiotics were prescribed for most patients [136 (80.5%)]. In multivariate analysis, septic shock at ICU admission [OR 5.52 (1.92–15.84)], the use of invasive MV [OR 12.74 (3.60–45.07)] and poor *Performance Status* [OR 3.00 (1.07–8.42)] were associated with increased hospital mortality.

**Conclusions:**

Severe pneumonia is associated with high mortality rates in cancer patients. A relatively low rate of MR pathogens is observed and severity of illness and organ dysfunction seems to be the best predictors of outcome in this population.

## Introduction

Pneumonia is the most frequent type of infection in cancer patients. Severe pneumonia accounts for approximately half of all cases of septic shock and is associated with exceedingly high mortality.[1,2]^,^ [3,4] Malignancy is a risk factor in the development of pneumonia and the co-existence of cancer is associated with increased severity of illness. [5,6]^,^ [7,8] Cancer patients often attend a hospital for anticancer treatments such as surgery, chemo- and radiation-therapy and, according to the current American Thoracic Society (ATS)/ Infectious Diseases Society of America (IDSA) guidelines, an event of pneumonia diagnosed outside the hospital setting in these patients is classified as healthcare-associated pneumonia (HCAP). [9,10] Several observational studies reported higher mortality rates among HCAP patients as compared to community-acquired pneumonia (CAP) patients because the former are at higher risk of inadequate initial antimicrobial treatment due to the presence of multiresistant (MR) pathogens. [11–16] Such findings have encouraged authors to recommend broad spectrum antimicrobials aiming at MR pathogens in patients with HCAP in a similar approach as to those recommended to nosocomial pneumonia [10,12,17], [10,18], [10,19]. To date, the impact of the broad-spectrum antimicrobial approach remains controversial.[17,20]

The primary aims of this study were to describe a population of cancer patients with severe pneumonia (not acquired in the hospital setting) who required intensive care unit (ICU) admission and identify predictors of hospital mortality. Secondary aims included a comparison between patients classified as CAP and HCAP as well as a comparison between MR versus no MR populations, describing microbiological variables and outcomes.

## Patients and Methods

### Design and Setting

This was a secondary analysis of a prospective cohort of patients admitted to three ICUs in Brazil (two in referral exclusive cancer centers and one in a high-volume tertiary hospital with a dedicated cancer center) between January 2002 to October 2013. Local guidelines for the antimicrobial treatment of pneumonia were provided by infection control departments at each institution and constantly updated as based on serial evaluation of local microbiological patterns.

This study was strictly observational and did not interfere with clinical decisions related with patient care. The Ethics Committee of the coordinating Center approved the study (Pareceres CEP INCA N° 12/2001 and N° 10/2003) and the need for informed consent was waived. Local IRBs on the other two centers reviewed and approved the study.

### Selection of Participants, Data Collection and Definitions

During the study period, every adult patient (>18yrs) with a definite diagnosis of cancer and presenting with pneumonia not acquired in the hospital setting was evaluated upon ICU admission. Patients with recent diagnosis of cancer, receiving anticancer treatment and patients with cancer relapse are considered with active cancer and they were included in this study. Patients in complete remission for > 5 years were not considered. The diagnosis of pneumonia was based on the presence of selected clinical features (e.g., cough, fever, sputum production and pleuritic chest pain) and supported by imaging of the lung in the first 48h of hospital admission.[21,22] Microbiological confirmation was not a compulsory inclusion criterion for the study. Patients in complete remission (>5 years) and those with diagnosis of nosocomial pneumonia or ventilator-associated pneumonia (VAP) were excluded.

Sepsis, severe sepsis and septic shock were diagnosed according to the consensus definitions. [1,22] Data related to patient’s management and adherence to process of care measures were not collected. Demographic, clinical and laboratory data were collected using standardized case report forms during the first day of ICU including the Simplified Acute Physiology Score (SAPS) II, [3,10] the Sequential Organ Failure Assessment (SOFA) score, [5,23] comorbidities, *performance status* (PS) [Eastern Cooperative Oncology Group scale], [7,24] and cancer- and treatment-related data. The hematological malignancies were classified as high or low-grade. [9,10] Comorbidities were evaluated using the Charlson Comorbidity Index. [11,17] Neutropenia was defined as a neutrophil count below 500/mm^3^. Microbiological data included bacterial isolation in tracheal aspirate or bronchoalveolar lavage and blood culture, pathogen identification and antimicrobial sensitivity test, MR pathogen identification, empiric antibiotic treatment and the adherence to the ATS guidelines. [10,17,25] Adequate empiric antibiotic treatment was based in the sensitivity test of the identified bacteria. The sensitivity test was performed based in Clinical and Laboratory Standards Institute definitions [4,18]. ATS/IDSA *guidelines* adherence was based in the recommendations of empiric antimicrobial treatment for CAP and HCAP [10,26]^,^ [17,27]. MR pathogens were defined as non-susceptibility to at least one agent in three or more antimicrobial categories. [12,21] Vital status at hospital discharge was the main outcome of interest. ICU mortality rates were also assessed.

Patients were classified in two categories: CAP and HCAP. CAP was defined as pneumonia diagnosed in the first 48h of hospital admission based on the current criteria. [22,28] The presence of one of the following criteria classified the patients’ condition as HCAP: hospitalization in an acute care hospital for two or more days within 90 days of the infection; residence in a nursing-home or long-term care facility, intravenous antibiotic therapy, chemotherapy, or wound care within the past 30 days of the current infection, or attended a hospital or hemodialysis clinic. [10,29]

### Data processing and statistical analysis

Data entry was performed by two investigators (MS and LSR) and consistency was assessed by a rechecking procedure of a 10% random sample of patients. Data were screened in detail for missing information, implausible and outlying values. Standard descriptive statistics were used. Continuous variables were reported as median [25%-75% interquartile range, (IQR)]. Univariate and multivariate logistic regression analyses were used to identify factors associated with hospital mortality. The area under the receiver-operating characteristic curve (AROC) was used to assess the models’ discrimination [23,30]. Variables yielding P values <0.2 in the univariate analysis and those considered clinically relevant according to the current literature [12,24] were entered into the multivariate analyses. The SPSS 13.0 software package (Chicago, Illinois, USA) and Prism 3.0 (Graphpad, USA) were used for statistical analysis.

## Results

### Main characteristics of study population

During the study period, a total of 325 patients fulfilled the eligibility criteria and were evaluated. There were 229 (71%) patients with solid tumors and 96 (29%) patients with hematological malignancies. The main primary sites of solid tumors were head and neck 72 (22%), lung 38 (12%) and urogenital 27 (8%); 96 patients had hematological malignancies and were classified in high-grade 48 (15%) and low-grade 48 (15%).

Neutropenia was present in 35 (11%) patients at ICU admission. No patient presented prolonged neutropenia. Regarding supportive care, 75% of patients were in septic shock, 91% of patients needed ventilatory support, mainly (81%) invasive mechanical ventilation (MV). Renal replacement therapy (RRT) was employed in 27% patients. The patients’ main characteristics are depicted in Table 1.

**Table 1:** Univariate analysis of characteristics associated with hospital mortality of cancer patients admitted with pneumonia acquired out of hospital setting.

	All Patients n = 325 (100%)	Survivors n = 114 (35%)	Nonsurvivors n = 211 (65%)	***P***-value
**Age (years)**	66 (55.5–73)	64 (52.75–72)	67 (57–75)	0.307
**Male gender**	203 (63%)	70 (61%)	133 (63%)	0.811
***Performance status***				
**0–1**	174 (54%)	68 (60%)	106 (50%)	0.130
**2–4**	148 (46%)	45 (40%)	103 (49%)	
**Previous hospitalization** ^**1**^	106 (33%)	39 (34%)	67 (32%)	0.710
**Previous chemotherapy** ^**2**^	109 (34%)	35 (31%)	74 (35%)	0.462
**Previous radiation therapy** ^**3**^	35 (11%)	12 (11%)	23 (11%)	0.999
**Attended a hospital, nursing home or hemodialysis clinic**	49 (15%)	26 (23%)	23 (11%)	0.006
**Solid tumors**	229 (71%)	77 (68%)	152 (72%)	0.445
**Hematologic malignancy**	96 (30%)	37 (33%)	59 (28%)	
**Hospital LOS prior to ICU admission**	1 (0–2)	1 (0–2)	1 (0–2)	0.101
**Charlson comorbidity index (points)**	3 (2–6)	3 (2–5)	3 (2–6)	0.702
**Neutropenia**	35 (11%)	11 (10%)	24 (11%)	0.710
**Septic shock at ICU admission**	244 (75%)	63 (55%)	181 (86%)	<0.001
**SOFA on Day 1 (points)**	7 (5–10)	6 (3.75–8.25)	8 (6–11)	<0.001
**SAPS II (points)**	50 (38–61)	45 (34–54)	52 (43–65.5)	<0.001
**Ventilatory support category**				
**None**	28 (9%)	22 (19%)	6 (3%)	<0.001
**Noninvasive mechanical ventilation (NIV) only**	35 (11%)	24 (21%)	11 (5%)	<0.001
**NIV followed by MV**	50 (15%)	15 (13.2%)	35 (17%)	0.520
**Invasive MV only**	262 (81%)	68 (60%)	194 (92%)	<0.001
**RRT**	88 (27%)	11 (10%)	77 (37%)	<0.001
**Corticosteroids use 30 days before**	97 (30%)	34 (30%)	63 (30%)	0.999

*1- Previous hospitalization is defined when a patient was hospitalized in an acute care hospital for two or more days within 90 days of the infection*.

*2- Previous chemotherapy is defined as chemotherapy within the past 30 days of the current infection*.

*3- Previous radiation therapy is defined as radiation therapy within the past 30 days of the current infection*.

*Definition of abbreviations*: *LOS = length of stay; ICU = intensive care unit; NIV = noninvasive ventilation; SOFA score = sequential organ failure assessment score; SAPS score = simplified acute physiology score; RRT = renal replacement therapy*

### Microbiological characteristics

Microbiological confirmation was present in 169 (52%) of study population with a predominance of Gram negative bacteria [*n* = 99 (59%)]. There were 40 (24%) positive blood cultures and 150 (89%) bacterial isolation in tracheal aspiration sample or bronchoalveolar lavage. Simultaneous bacterial identification was present in blood culture and tracheal aspiration sample or bronchoalveolar lavage in 21 (12%) patients. The most frequently identified pathogens were methicillin-sensitive *S*. *aureus* (MSSA) [*n* = 42 (25%)], followed by *P*. *aeruginosa* [*n* = 41 (24%)] and *S*. *pneumoniae* [*n* = 21 (12%)]. MR pathogens represented 13.6% (*n* = 23) of patients. Detailed microbiological data is described in Table 2.

**Table 2:** Microbiological data according to survival of critically ill cancer patients with pneumonia.

	All Patients n = 325 (100%)	Survivors n = 114 (35%)	Nonsurvivors n = 211 (65%)	P Value
**Microbiological confirmation**	169 (52%)	53 (47%)	116 (55%)	0.163
**Adequate antibiotic therapy** ^**1**^	136 (81%)	45 (85%)	91 (78%)	0.405
**Positive blood culture**	40 (24%)	15 (28%)	25 (22%)	0.338
**Gram-negative bactéria**	99 (59%)	36 (68%)	63 (54%)	0.130
***Pseudomonas aeruginosa***	41 (24%)	10 (19%)	31 (27%)	0.335
***Klebsiella pneumoniae***	15 (9%)	4 (8%)	11 (10%)	0.779
**Gram-positive bactéria**	69 (41%)	22 (42%)	47 (41%)	0.999
***Staphylococcus aureus***	42 (25%)	12 (23%)	30 (26%)	0.705
***Streptococcus pneumoniae***	21 (12%)	10 (19%)	11 (10%)	0.129
**MR Pathogens** ^**2**^	23 (14%)	6 (11%)	17 (15%)	0.636
**MRSA**	11 (7%)	3 (6%)	8 (7%)	0.999
**ATS Guideline adherence** ^**3**^	53 (16%)	25 (22%)	28 (13%)	0.058
**Macrolide use**	66 (20%)	30 (26%)	36 (17%)	0.060
**Atypical pathogen coverage**	116 (36%)	52 (46%)	64 (30%)	0.007
**Only quinolone use**	50 (15%)	22 (19%)	28 (13%)	0.197
**Number of antimicrobial agentes**				
**1**	154 (47%)	45 (40%)	109 (52%)	0.037
**2**	126 (39%)	49 (43%)	77 (37%)	
**> 2**	44 (14%)	17 (15%)	27 (13%)	

*1- Adequate empiric antibiotic treatment was based in the sensitivity test of the identified bacteria*.

*2- The MR pathogens were defined as non-susceptibility to at least one agent in three or more antimicrobial categories*. [6,21]

*3- ATS/IDSA guidelines adherence was based in definitions of empiric antimicrobial treatment for CAP and HCAP*. [8,10], [10,17]

The evaluation of antibiotic therapy based on microbiological identification (and *in vitro* susceptibility testing) demonstrated that adequate antibiotic therapy was prescribed in the majority of patients [136 (81%)]. However, adherence to CAP and HCAP ATS/IDSA guidelines [10,19], [13,17] was observed in only 53 (16%) patients. In the HCAP group, the most common reasons for non-adherence to the ATS/IDSA *guidelines* were the absence of double therapy (addition of amikacin or quinolones) [*n* = 174 (54%)] and lack of coverage for methicillin-resistant S. aureus (MRSA) [*n* = 134 (41%)] or *Pseudomonas sp* [*n* = 32 (10%)].

We performed a comparison between patients where MR pathogens were identified (*n* = 23) and those without MR pathogens (*n* = 146). Demographic characteristics, type of cancer, need for life-sustaining therapies and clinical outcomes were comparable between the two groups. There were no significant differences regarding ATS/IDSA *guidelines* adherence in these two groups (MR patients 27% vs. no MR 14%, *P* = 0.358). Detailed comparisons between MR patients and no MR patients are provided in Tables 3 and 4.

**Table 3:** Demographic and clinical variables of patients admitted in the ICU with pneumonia according to the presence of MR pathogens.

	MR patients n = 23 (14%)	No MR patients n = 146 (86%)	P Value
**Age (years)**	72 (59–77)	67 (57–73)	0.126
**Male gender**	16 (70%)	89 (61%)	0.494
***Performance Status***			
**0–1**	8 (35%)	75 (51%)	0.179
**2–4**	15 (65%)	69 (47%)	
**Solid tumors**	15 (65%)	102 (70%)	0.635
**Hematologic malignancy**	8 (35%)	44 (30%)	
**Hospital LOS prior ICU (days)**	1 (0–2)	1 (0–2)	0.901
**Charlson comorbidity**	2 (2–6)	3 (2–5)	0.484
**Neutropenia**	3 (13%)	13 (9%)	0.460
**Septic shock at ICU admission**	19 (83%)	121 (83%)	0.999
**SOFA D1 – points**	9 (6–11)	7 (5–11)	0.247
**SAPS II – points**	52 (46–63)	51 (41–61)	0.576
**Ventilatory support category**			
**None**	0 (0%)	5 (4%)	0.999
**Exclusive NIV**	1 (4%)	13 (9%)	0.695
**NIV followed by MV**	4 (17%)	29 (20%)	0.999
**MV**	22 (96%)	128 (88%)	0.476
**RRT**	10 (44%)	45 (31%)	0.239
**Corticosteroids use 30 days before**	7 (30%)	38 (26%)	0.622
**ICU mortality**	13 (57%)	74 (51%)	0.658
**Hospital mortality**	17 (74%)	99 (68%)	0.636
**ICU LOS (days)**	14 (9–25)	10 (4–18)	0.052
**Hospital LOS (days)**	27 (11–34)	16 (8–34.25)	0.550

*Definition of abbreviations*: *LOS = length of stay; ICU = intensive care unit; NIV = noninvasive ventilation; SOFA score = sequential organ failure assessment score; SAPS score = simplified acute physiology score; RRT = renal replacement therapy*

**Table 4:** Microbiological data of patients admitted in the ICU with pneumonia and classified according to the presence of MR pathogens.

	MR patients n = 23 (14%)	No MR patients n = 146 (86%)	P Value
**Adequate antibiotic therapy** ^**1**^	7 (30%)	125 (86%)	<0.001
**Positive blood culture**	5 (22%)	35 (24%)	0.999
**Gram negative**	13 (57%)	84 (58%)	0.999
***Pseudomonas aeruginosa***	3 (13%)	38 (26%)	0.294
***Klebsiella pneumoniae***	3 (13%)	12 (8%)	0.434
**Gram positive**	13 (57%)	56 (38%)	0.114
***Staphylococcus aureus***	13 (57%)	29 (20%)	<0.001
***Streptococcus pneumoniae***	0 (0%)	21 (14%)	0.081
**ATS Guideline adherence** ^**2**^	5 (22%)	21 (14%)	0.358
**Number of antimicrobial drugs**			
**1**	14 (61%)	73 (50%)	0.375
**2**	7 (30%)	54 (37%)	
**> 2**	2 (9%)	19 (13%)	

*1- Adequate empiric antibiotic treatment was based in the sensitivity test of the identified bacteria*.

*2- ATS/IDSA guidelines adherence was based in definitions of empiric antimicrobial treatment for CAP and HCAP*. [10,12–16], [12,17]

### Outcomes analysis

The ICU and hospital mortality rates were 46% and 65%, respectively. Higher severity of illness and need for life-sustaining therapies as vasopressors use, invasive MV and need of RRT were observed in the group of nonsurvivors. Adequate antibiotic therapy was comparable in both groups. Atypical pathogen coverage was more frequently prescribed in survivors than in non-survivors (46% vs. 30%, *P* = 0.007]. Factors associated with hospital mortality in a univariate analysis were described in Table 1.

When we analysed the subgroup of patients with microbiological confirmation, we observed more frequence of good PS (0–1) in the group of survivors [Surv 33 (62%) vs 50 (43%) p = 0.031]. However higher severity of illness and need for life-sustaining therapies as vasopressors use, invasive MV and need of RRT were more frequent in nonsurvivors. Detailed comparison of patients with microbiological confirmation was described in S7 and S8 Tables.

In multivariate analysis, septic shock at ICU admission, the use of invasive MV and poor PS were associated with increased hospital mortality. A multivariate analysis of predictors of hospital mortality was described in Table 5.

**Table 5:** Multivariate analysis of predictors of hospital mortality in all patients admitted to the ICU with pneumonia and cancer (n = 325).

Variables	Coefficients	Odds Ratio (95% CI)	p value
**Invasive Mechanical Ventilation in the ICU**	2.545	12.74 (3.60–45.07)	<0. 001
**Septic Shock at ICU admission**	1.708	5.52 (1.92–15.85)	0.002
***Performance Status***	1.100	3.00 (1.07–8.42)	0.037
**Age**	0.016	1.02 (0.98–1.05)	0.315
**Charlson Index**	0.063	1.07 (0.83–1.36)	0.617
**SOFA D1**	0.044	1.05 (0.91–1.20)	0.538
**CAP vs HCAP**	0.888	2.43 (0.89–6.61)	0.082
**Macrolides use**	-0.203	0.82 (0.23–2.87)	0.752
**MR pathogens**	0.222	1.25 (0.17–9.31)	0.829
**Adequate antibiotic therapy** ^**1**^	-0.788	0.45 (0.08–2.75)	0.391
**Constant**	-4.0986		

*1- Adequate empiric antibiotic treatment was based in the sensitivity test of the identified bacteria*.

*Definition of abbreviations*: *CAP – community-acquired pneumonia; HCAP – healthcare-associated pneumonia; MR – Multiresistant; RRT = renal replacement therapy; SAPS score = simplified acute physiology score; SOFA score = sequential organ failure assessment score*.

*Area under receiver operating characteristic curve*, *0*.*822 (95% CI*, *0*.*746–0*.*883); Hosmer-Lemeshow goodness-of-fit (χ* ^*2*^ = *2*.*0925 p = 0*.*5534)*

### Comparisons between HCAP and CAP patients

The study population was classified as CAP [*n* = 132 (41%)] and HCAP [*n* = 193 (59%)]. The most frequent criteria for HCAP classification were: previous chemotherapy [*n* = 109 (57%)], prior hospitalization [*n* = 106 (55%)], attended a hospital, nursing home or hemodialysis clinic [*n* = 49 (25%)] and previous radiation therapy [*n* = 35 (18%)]. Patients with HCAP had a higher frequency of neutropenia at ICU admission and previous corticosteroids use as compared to those with CAP.

No significant differences in microbiological characteristics were found when CAP (*n* = 132) and HCAP (*n* = 193) were compared (S2 Table). Adherence to ATS guidelines was reduced in both groups but it was markedly lower in HCAP patients [CAP, 38% vs. HCAP, 1.6%, *P*<0.001]. However adequate antibiotic therapy was comparable between CAP and HCAP 81% vs. 80%, *P* = 0.99.

No significant differences were observed when we compared hospital mortality [64% vs. 65%, *P* = 0.906] and hospital length of stay [16 (9–35) vs. 15 (8–29), *P* = 0.205] between CAP and HCAP. Detailed characteristics of CAP and HCAP patients separately are provided (S3 and S4 Tables).

## Discussion

The present study that evaluated clinical outcomes, described microbiological characteristics and compared outcomes of CAP and HCAP in critically ill cancer patients with severe pneumonia. Epidemiological studies describing clinical characteristics of this subpopulation are scarce [2], [31]. For the overall population, factors such as invasive mechanical ventilation use, septic shock in the ICU admission and poor PS were independently associated with hospital mortality. Based on ATS/IDSA *guidelines*, we compared the clinical and microbiological characteristics of CAP and HCAP cancer patients admitted in the ICU and the clinical and microbiological characteristics as well as hospital mortality were similar in both groups.

We were not able to identify substantial differences between patients classified as CAP and HCAP in our study. Recently, a case-control study presented similar findings when compared the characteristics and outcomes of HCAP and CAP patients at hospital level.[25,26] Interestingly, in the present study, cancer patients with pneumonia presented characteristics when compared to the current literature of general CAP patients that impose a distinct management. The most common pathogens identified in our population were MSSA and *P*. *aeruginosa*, which were not the most frequent in contemporaneous cohorts of general patients with severe CAP [4,25]. Interestingly, in the present study *Streptococcus pneumoniae* was only isolated in 12% of patients with microbiological confirmation. Additionally, these findings of cancer patients are not comparable to other groups of HCAP patients such as previous hospitalization, patients from nursing home or from hemodialysis clinic [26,32], [27] Although an increasing prevalence of resistant organisms in community-acquired pneumonia patients has been reported in some studies, [12,33]^,^ [28,34] it was not confirmed in other recent cohorts [29,35,36], [2,30]. Even though the present study deals with cancer patients, it confirmed a low prevalence of MR bacteria (13.6%) with no influence in clinical outcomes [Survivors 6 (11.3%) vs. Nonsurvivors 17 (14.7%), p = 0.636]. High incidence of MR pathogens has been described in epidemiological studies including patients with healthcare-associated pneumonia [12,37]^,^ [19]^,^ [13], [26]. Additionally, in our study, the presence of MR pathogens was not an independent predictor factor associated with hospital mortality. The poor outcomes observed in patients with HCAP can also be related to the presence of more comorbidities and poor functional status. [25], [32]

The lack of association between MR pathogens and mortality rates was described in other two studies that included patients hospitalized (but mainly non-ICU patients) with pneumonia, with low hospital mortality rates (< 15%). [27]^,^ [33]

The present study has some limitations. First, this was a strictly observational study and although the centers have a high case volume of cancer patients, specific protocols for the investigation and care of patients with pneumonia were not designed and implemented in our study. Therefore, we did not employ systematically other microbiological investigation methods (eg serology, PCR assays, urinary antigens) to establish the etiologic diagnosis of pneumonia, which limited our ability to identify pathogens such as viruses and atypical pathogens. Additionally, we did not collect data on process of care measures, other than adherence to antimicrobial guidelines, although we acknowledge that these may be important contributors to clinical outcomes [34]. Additionally, the number of patients with hematological malignancies is relatively low in our study. However, this is in accordance with recent multicenter studies that demonstrate that solid tumors are the most frequent type of malignancy in critically ill cancer patients [35,36]. Nonetheless, it is possible that for this reason the present study may be underpowered for the detection of opportunistic pathogens. Moreover we acknowledge the high hospital mortality (64.9%) in the present study. However, comparable mortality rates were observed in recent study with critically ill cancer patients admitted to the ICU with pneumonia and sepsis. [2], [37], [2,31] Another important consideration was that our period of inclusion is long (2002–2013) and we acknowledge that the improvement in the process of care was observed in the last decade may have occurred for patients with cancer and sepsis. [4,38] To clarify the influence of two distinct inclusion periods, we performed a comparison between 2002–2005 and 2006–2013 and no major differences were observed in ICU and hospital mortality or length of stay [S9 and S10 Tables]. To end, we included in the study the following outcomes: ICU and hospital mortality and ICU and hospital length of stay (LOS). MV-free days can be an important outcome for patients with sever CAP but this was beyond the scope of the study. Our study focused in outcome predictors at ICU admission.

In conclusion, we believe that cancer patients are a distinct group of patients with pneumonia and that a classification of HCAP or CAP according to ATS criteria does not add to risk assessment or to guidance of antimicrobial therapy. In these patients, a relatively low rate of MR pathogens is observed and severity of illness and organ dysfunction seems to be the best predictors of outcome in this population.

## Supporting Information

S1 Table Clinical characteristics of patients admitted to ICU with pneumonia and classified as Community-Acquired Pneumonia (CAP) or Healthcare-Associated Pneumonia (HCAP). Definition of abbreviations: CAP = community-acquired pneumonia; HCAP = healthcare-associated pneumonia; LOS = length of stay; ICU = intensive care unit; NIV = noninvasive ventilation; SOFA score D1 = sequential organ failure assessment score in first day at ICU; SAPS II score = simplified acute physiology score; RRT = renal replacement therapy.
(DOCX)

S2 Table Microbiological data according to the ATS/IDSA classification of Community-Acquired Pneumonia(CAP) and Healthcare-Associated Pneumonia (HCAP). *1- Adequate empiric antibiotic treatment was based in the sensitivity test of the identified bacteria*. *2- The MR pathogens were defined as non-susceptibility to at least one agent in three or more antimicrobial categories*. *{Magiorakos*:*2012be} 3- ATS/IDSA guidelines adherence was based in definitions of empiric antimicrobial treatment for CAP and HCAP*. *{AmericanThoracicSociety*:*2005kw}*, *{Mandell*:*2007ik}*. Definition of abbreviations: ATS = American Thoracic Society; CAP = Community Acquired Pneumonia; HCAP = Healthcare-associated Pneumonia; MR = Multiresistant; MRSA = Methicilin-resistant *Staphylococcus aureus*.
(DOCX)

S3 Table Demographic and clinical variables of Community-Acquired Pneumonia (CAP) patients and characteristics associated with hospital mortality. Definition of abbreviations: CAP = community-acquired pneumonia; LOS = length of stay; ICU = intensive care unit; NIV = noninvasive ventilation; SOFA score D1 = sequential organ failure assessment score in first day at ICU; SAPS II score = simplified acute physiology score; RRT = renal replacement therapy.
(DOCX)

S4 Table Demographic and clinical variables of Healthcare-Associated Pneumonia (HCAP) patients and characteristics associated with hospital mortality. Definition of abbreviations: HCAP = healthcare-associated pneumonia; LOS = length of stay; ICU = intensive care unit; NIV = noninvasive ventilation; SOFA score D1 = sequential organ failure assessment score in first day at ICU; SAPS II score = simplified acute physiology score; RRT = renal replacement therapy.
(DOCX)

S5 Table Demographic and clinical variables of patients admitted in the ICU with pneumonia according to the type of cancer. Definition of abbreviations: LOS = length of stay; ICU = intensive care unit; NIV = noninvasive ventilation; SOFA score D1 = sequential organ failure assessment score in first day at ICU; SAPS II score = simplified acute physiology score; RRT = renal replacement therapy.
(DOCX)

S6 Table Microbiological data of patients admitted in the ICU with pneumonia and classified according to the type of cancer. *1- Adequate empiric antibiotic treatment was based in the sensitivity test of the identified bacteria*. *2- The MR pathogens were defined as non-susceptibility to at least one agent in three or more antimicrobial categories*. {Magiorakos:2012be}. *3- ATS/IDSA guidelines adherence was based in definitions of empiric antimicrobial treatment for CAP and HCAP*. {AmericanThoracicSociety:2005kw}, {Mandell:2007ik}. Definition of abbreviations: ATS = American Thoracic Society; MR = Multiresistant; MRSA = Methicilin-resistant *Staphylococcus aureus*.
(DOCX)

S7 Table Microbiological data according to survival of critically ill cancer patients admitted in the ICU with pneumonia with microbiological confirmation. *1- Adequate empiric antibiotic treatment was based in the sensitivity test of the identified bacteria*. *2- The MR pathogens were defined as non-susceptibility to at least one agent in three or more antimicrobial categories*. ^22^ *3- ATS/IDSA guidelines adherence was based in definitions of empiric antimicrobial treatment for CAP and HCAP*. ^5^, ^20^ Definition of abbreviations: ATS = American Thoracic Society; MR = Multiresistant; MRSA = Methicilin-resistant *Staphylococcus aureus*.
(DOCX)

S8 Table Demographic and clinical variables of patients admitted in the ICU with pneumonia with microbiological confirmation. *1- Previous hospitalization is defined when a patient was hospitalized in an acute care hospital for two or more days within 90 days of the infection*. *2- Previous chemotherapy is defined as chemotherapy within the past 30 days of the current infection*. *3- Previous radiation therapy is defined as radiation therapy within the past 30 days of the current infection*. Definition of abbreviations: LOS = length of stay; ICU = intensive care unit; NIV = noninvasive ventilation; SOFA score = sequential organ failure assessment score; SAPS score = simplified acute physiology score; RRT = renal replacement therapy
(DOCX)

S9 Table Demographic and clinical variables of patients admitted in the ICU with pneumonia and classified according to inclusion period 2002–2005 and 2006–2013. Definition of abbreviations: LOS = length of stay; ICU = intensive care unit; NIV = noninvasive ventilation; SOFA score D1 = sequential organ failure assessment score in first day at ICU; SAPS II score = simplified acute physiology score; RRT = renal replacement therapy.
(DOCX)

S10 Table Microbiological data of patients admitted in the ICU with pneumonia and classified according to inclusion period 2002–2005 and 2006–2013. *1- Adequate empiric antibiotic treatment was based in the sensitivity test of the identified bacteria*. *2- The MR pathogens were defined as non-susceptibility to at least one agent in three or more antimicrobial categories*. *{Magiorakos*:*2012be}*. *3- ATS/IDSA guidelines adherence was based in definitions of empiric antimicrobial treatment for CAP and HCAP*. *{AmericanThoracicSociety*:*2005kw}*, *{Mandell*:*2007ik}*. Definition of abbreviations: ATS = American Thoracic Society; MR = Multiresistant; MRSA = Methicilin-resistant *Staphylococcus aureus*.
(DOCX)
