# Supplementary material for: Clinical Outcomes and Microbiological Characteristics of Severe Pneumonia in Cancer Patients: A Prospective Cohort Study
Source: PLoS One. 2015 Mar 24;10(3):e0120544. doi: 10.1371/journal.pone.0120544 (PMC4372450; doi:10.1371/journal.pone.0120544)
Supplement: S1 Table — Definition of abbreviations: CAP = community-acquired pneumonia; HCAP = healthcare-associated pneumonia; LOS = length of stay; ICU = intensive care unit; NIV = noninvasive ventilation; SOFA score D1 = sequential organ failure assessment score in first day at ICU; SAPS II score = simplified acute physiology score; RRT = renal replacement therapy. (DOCX) [file pone.0120544.s001.docx]

**S1 Table – Clinical characteristics of patients admitted to ICU with pneumonia and classified as Community-Acquired Pneumonia (CAP) or Healthcare-Associated Pneumonia (HCAP)**

|  | **CAP**  **n= 132 (41%)** | **HCAP**  **n= 193**  **(59%)** | **P Value*** |
| --- | --- | --- | --- |
| **Age (years)** | 67  (58 – 75) | 64  (54 – 73) | 0.167 |
| **Male gender** | 94  (71%) | 109  (57%) | 0.008 |
| ***Performance Status*** |  |  |  |
| **0-1** | 69  (52%) | 105  (54%) | 0.735 |
| **2-4** | 62  (47%) | 86  (45%) |  |
| **Solid tumors** | 100  (76%) | 129  (67%) | 0.107 |
| **Hematological malignancies** | 32  (24%) | 64  (33%) |  |
| **Hospital LOS prior ICU (days)** | 1  (0–2) | 1  (0– 2) | 0.381 |
| **Charlson comorbidity Index (points)** | 3  (2–4) | 3  (2– 6) | 0.050 |
| **Neutropenia** | 6  (5%) | 29  (15%) | 0.003 |
| **Septic shock at ICU admission** | 108  (82%) | 136  (71%) | 0.026 |
| **SOFA D1 (points)** | 7  (5 – 10) | 7  (5 – 10) | 0.910 |
| **SAPS II (points)** | 49  (40 – 59) | 50  (38 – 61.75) | 0.480 |
| **Ventilatory support category** |  |  |  |
| **None** | 4  (3%) | 18  (9%) | 0.041 |
| **NIV, only** | 13  (10%) | 28  (15%) | 0.237 |
| **NIV followed by MV** | 23  (17%) | 27  (14%) | 0.436 |
| **MV, only** | 115  (87%) | 147  (76%) | 0.015 |
| **RRT** | 37  (28%) | 51  (26%) | 0.800 |
| **Corticosteroids use 30 days before hospital admission** | 19  (14%) | 78  (40%) | <0.001 |
| **ICU mortality** | 52  (39%) | 97  (50%) | 0.055 |
| **Hospital mortality** | 85  (64%) | 126  (65%) | 0.906 |
| **ICU LOS (days)** | 10  (4–17) | 7  (3–14) | 0.057 |
| **Hospital LOS (days)** | 16  (9–34.75) | 15  (8–29) | 0.205 |

Definition of abbreviations: CAP= community-acquired pneumonia; HCAP= healthcare-associated pneumonia; LOS= length of stay; ICU= intensive care unit; NIV= noninvasive ventilation; SOFA score D1= sequential organ failure assessment score in first day at ICU; SAPS II score= simplified acute physiology score; RRT= renal replacement therapy

**S2 Table – Microbiological data according to the ATS/IDSA classification of Community-Acquired Pneumonia(CAP) and Healthcare-Associated Pneumonia (HCAP)**

|  | **CAP**  **n= 132**  **(41%)** | **HCAP**  **n= 193**  **(59%)** | **P Value*** |
| --- | --- | --- | --- |
| **Adequate antibiotic therapy^1^** | 58  (81%) | 78  (80%) | 0.999 |
| **Positive blood culture** | 16  (22%) | 24  (25%) | 0.855 |
| **Gram negative** | 50  (69%) | 49  (50%) | 0.012 |
| ***Pseudomonas aeruginosa*** | 19  (26%) | 22  (22%) | 0.589 |
| ***Klebsiella pneumoniae*** | 6  (8%) | 9  (9%) | 0.999 |
| **Gram positive** | 28  (39%) | 41  (42%) | 0.753 |
| ***Staphylococcus aureus*** | 15  (21%) | 27  (28%) | 0.370 |
| ***Streptococcus pneumoniae*** | 10  (14%) | 11  (11%) | 0.642 |
| **MR Pathogens^2^** | 9  (13%) | 14  (14%) | 0.823 |
| **MRSA** | 3  (4%) | 8  (8%) | 0.359 |
| **ATS Guidelines adherence^3^** | 50  (38%) | 3  (2%) | <0.001 |
| **Macrolide use** | 25  (19%) | 41  (21%) | 0.675 |
| **Atypical pathogen coverage** | 57  (43%) | 59  (31%) | 0.025 |
| **Only quinolone use** | 24  (18%) | 6  (3%) | <0.001 |
| **Number of antimicrobial agents** |  |  |  |
| **1** | 75  (57%) | 79  (41%) | 0.007 |
| **2** | 44  (33%) | 82  (43%) |  |
| **> 2** | 12  (9%) | 32  (17%) |  |

*1- Adequate empiric antibiotic treatment was based in the sensitivity test of the identified bacteria.*

*2- The MR pathogens were defined as non-susceptibility to at least one agent in three or more antimicrobial categories. {Magiorakos:2012be}*

*3- ATS/IDSA guidelines adherence was based in definitions of empiric antimicrobial treatment for CAP and HCAP. {AmericanThoracicSociety:2005kw}, {Mandell:2007ik}*

Definition of abbreviations: ATS= American Thoracic Society; CAP= Community Acquired Pneumonia; HCAP= Healthcare-associated Pneumonia; MR= Multiresistant; MRSA= Methicilin-resistant *Staphylococcus aureus.*

**S3 Table – Demographic and clinical variables of Community-Acquired Pneumonia (CAP) patients and characteristics associated with hospital mortality**

|  | **All CAP Patients**  **n= 132(100%)** | **Survivors**  **n= 47**  **(36%)** | **Nonsurvivors**  **n= 85**  **(64%)** | **P Value*** |
| --- | --- | --- | --- | --- |
| **Age (years)** | 67  (58– 75) | 66  (58 – 74) | 68  (57.5 – 75) | 0.992 |
| **Male gender** | 94  (71%) | 33  (70%) | 61  (72%) | 0.844 |
| **Performance Status*** |  |  |  |  |
| **0-1** | 69  (52%) | 30  (64%) | 39  (46%) | 0.068 |
| **2-4** | 62  (47%) | 17  (36%) | 45  (53%) |  |
| **Solid tumors** | 100  (76%) | 36  (77%) | 64  (75%) | 0.999 |
| **Hematological malignancies** | 32  (24%) | 11  (23%) | 21  (25%) |  |
| **LOS prior ICU (days)** | 1  (0 – 2) | 1  (0 – 2) | 1  (0 – 2) | 0.621 |
| **Charlson comorbidity Index (points)** | 3  (2 – 4) | 3  (2 – 4) | 3  (2 – 4) | 0.278 |
| **Neutropenia** | 6  (5%) | 1  (2%) | 5  (6%) | 0.421 |
| **Septic shock at ICU admission** | 108  (82%) | 31  (66%) | 77  (91%) | <0.001 |
| **SOFA D1 (points)** | 7  (5 – 10) | 6  (4 – 9) | 8  (5 – 10.5) | 0.013 |
| **SAPS II (points)** | 49  (40 – 59) | 44  (35 – 53) | 52  (44 – 65) | <0.001 |
| **Ventilatory support category** |  |  |  |  |
| **None** | 4  (3%) | 3  (6%) | 1  (2%) | 0.129 |
| **NIV, only** | 13  (10%) | 11  (23%) | 2  (2%) | <0.001 |
| **NIV followed by MV** | 23  (17%) | 7  (15%) | 16  (19%) | 0.638 |
| **MV, only** | 115  (87%) | 33  (70%) | 82  (97%) | <0.001 |
| **RRT** | 37  (28%) | 5  (11%) | 32  (38%) | <0.001 |
| **Corticosteroids use 30 days before hospital admission** | 19  (14%) | 8  (17%) | 11  (13%) | 0.610 |

Definition of abbreviations: CAP= community-acquired pneumonia; LOS= length of stay; ICU= intensive care unit; NIV= noninvasive ventilation; SOFA score D1= sequential organ failure assessment score in first day at ICU; SAPS II score= simplified acute physiology score; RRT= renal replacement therapy

**S4 Table – Demographic and clinical variables of Healthcare-Associated Pneumonia (HCAP) patients and characteristics associated with hospital mortality**

|  | **All HCAP Patients**  **n= 193 (100%)** | **Survivors**  **n=67**  **(35%)** | **Nonsurvivors**  **n= 126**  **(65%)** | **P Value*** |
| --- | --- | --- | --- | --- |
| **Age (years)** | 64  (54 – 73) | 63  (51 – 72) | 65  (57–74) | 0.158 |
| **Male gender** | 109  (57%) | 37  (55%) | 72  (57%) | 0.879 |
| **Performance Status*** |  |  |  |  |
| **0-1** | 105  (54%) | 38  (57%) | 67  (53%) | 0.652 |
| **2-4** | 86  (45%) | 28  (42%) | 58  (46%) |  |
| **Previous Hospitalization^1^** | 106  (55%) | 39  (58%) | 67  (53%) | 0.545 |
| **Previous Chemotherapy^2^** | 109  (57%) | 35  (52%) | 74  (59%) | 0.446 |
| **PreviousRadiation Therapy^3^** | 35  (18%) | 12  (18%) | 23  (18%) | 0.999 |
| **Attended a hospital, nursing home or hemodialysis clinic** | 49  (25%) | 26  (39%) | 23  (18%) | 0.003 |
| **Solidtumors** | 129  (67%) | 41  (61%) | 88  (70%) | 0.262 |
| **Hematological malignancies** | 64  (33%) | 26  (39%) | 38  (30%) |  |
| **LOS prior ICU (days)** | 1  (0–2) | 1  (0–2) | 1  (0–2) | 0.098 |
| **Charlson comorbidity Index (points)** | 3  (2–6) | 3  (2–6) | 3  (2–6) | 0.212 |
| **Neutropenia** | 29  (15%) | 10  (15%) | 19  (15%) | 0.999 |
| **Septic shock at ICU admission** | 136  (71%) | 32  (48%) | 104  (83%) | <0.001 |
| **SOFA D1 (points)** | 7  (5–10) | 6  (3 – 8) | 8  (6–11) | <0.001 |
| **SAPS II (points)** | 50  (38–61.75) | 45  (34 – 56) | 53  (42.5–67) | <0.001 |
| **Ventilatory support category** |  |  |  |  |
| **None** | 18  (9%) | 13  (19%) | 5  (4%) | <0.001 |
| **NIV, only** | 28  (15%) | 19  (28%) | 9  (7%) | <0.001 |
| **NIV followed by MV** | 27  (14%) | 8  (12%) | 19  (15%) | 0.665 |
| **MV, only** | 147  (76%) | 35  (52%) | 112  (89%) | <0.001 |
| **RRT** | 51  (26%) | 6  (9%) | 45  (36%) | <0.001 |
| **Corticosteroids use 30 days before hospital admission** | 78  (40%) | 26  (39%) | 52  (42%) | 0.760 |

Definition of abbreviations: HCAP= healthcare-associated pneumonia; LOS= length of stay; ICU= intensive care unit; NIV= noninvasive ventilation; SOFA score D1= sequential organ failure assessment score in first day at ICU; SAPS II score= simplified acute physiology score; RRT= renal replacement therapy

**S5 Table – Demographic and clinical variables of patients admitted in the ICU with pneumonia according to the type of cancer**

|  | **Solid tumors**  **n= 229**  **(71%)** | **Hematological malignancies**  **n= 96**  **(29%)** | **P Value*** |
| --- | --- | --- | --- |
| **Age (years)** | 66  (57.5–74) | 64.5  (45–72.75) | 0.030 |
| **Male gender** | 144  (63%) | 59  (62%) | 0.803 |
| ***Performance Status*** |  |  |  |
| **0-1** | 118  (52%) | 56  (58%) | 0.275 |
| **2-4** | 109  (48%) | 34  (35%) |  |
| **Hospital LOS prior ICU (days)** | 1  (0–2) | 1  (0–2) | 0.864 |
| **Charlson comorbidity** | 3  (2–6) | 3  (2–4) | <0.001 |
| **Neutropenia** | 12  (5%) | 23  (24%) | <0.001 |
| **Septic shock at ICU admission** | 168  (73%) | 76  (79%) | 0.326 |
| **SOFA D1 – points** | 7  (4–10) | 8  (6–11) | 0.006 |
| **SAPS II – points** | 49  (37–59) | 54  (45–66) | 0.002 |
| **Ventilatory support category** |  |  |  |
| **None** | 13  (6%) | 9  (9%) | 0.233 |
| **Exclusive NIV** | 29  (13%) | 12  (13%) | 0.999 |
| **NIV followed by MV** | 36  (16%) | 14  (15%) | 0.867 |
| **MV** | 187  (82%) | 75  (78%) | 0.539 |
| **RRT** | 53  (23%) | 35  (37%) | 0.020 |
| **Corticosteroids use 30 days before** | 53  (23%) | 44  (46%) | <0.001 |
| **ICU mortality** | 101  (44%) | 48  (50%) | 0.393 |
| **Hospital mortality** | 152  (66%) | 59  (62%) | 0.445 |
| **ICU LOS (days)** | 8  (3.5–15.5) | 9  (3–15.75) | 0.864 |
| **Hospital LOS (days)** | 14  (8–31) | 17  (8–34.75) | 0.709 |

Definition of abbreviations: LOS= length of stay; ICU= intensive care unit; NIV= noninvasive ventilation; SOFA score D1= sequential organ failure assessment score in first day at ICU; SAPS II score= simplified acute physiology score; RRT= renal replacement therapy

**S6 Table - Microbiological data of patients admitted in the ICU with pneumonia and classified according to the type of cancer**

|  | **Solid tumors**  **n= 229**  **(71%)** | **Hematological**  **malignancies**  **n= 96**  **(29%)** | **P Value*** |
| --- | --- | --- | --- |
| **Adequate antibiotic therapy^1^** | 92  (79%) | 44  (85%) | 0.408 |
| **Positive blood culture** | 27  (23%) | 13  (25%) | 0.845 |
| **Gram negative** | 71  (61%) | 28  (54%) | 0.499 |
| ***Pseudomonas aeruginosa*** | 27  (23%) | 14  (27%) | 0.698 |
| ***Klebsiella pneumoniae*** | 10  (9%) | 5  (10%) | 0.778 |
| **Gram positive** | 46  (39%) | 23  (44%) | 0.612 |
| ***Staphylococcus aureus*** | 30  (26%) | 12  (23%) | 0.848 |
| ***Streptococcus pneumoniae*** | 13  (11%) | 8  (15%) | 0.455 |
| **MR Pathogens^2^** | 15  (13%) | 8  (15%) | 0.635 |
| **MRSA** | 7  (6%) | 4  (8%) | 0.739 |
| **ATS Guideline adherence^3^** | 39  (17%) | 14  (15%) | 0.626 |
| **Macrolide use** | 41  (18%) | 25  (26%) | 0.099 |
| **Atypical pathogen coverage** | 81  (35%) | 35  (37%) | 0.899 |
| **Only quinolone use** | 47  (21%) | 5  (5%) | <0.001 |
| **Number of antimicrobial drugs** |  |  |  |
| **1** | 115  (50%) | 39  (41%) | 0.144 |
| **2** | 89  (39%) | 37  (39%) |  |
| **> 2** | 24  (11%) | 20  (21%) |  |

*1- Adequate empiric antibiotic treatment was based in the sensitivity test of the identified bacteria.*

*2- The MR pathogens were defined as non-susceptibility to at least one agent in three or more antimicrobial categories.* {Magiorakos:2012be}

*3- ATS/IDSA guidelines adherence was based in definitions of empiric antimicrobial treatment for CAP and HCAP.* {AmericanThoracicSociety:2005kw}*,* {Mandell:2007ik}

Definition of abbreviations: ATS= American Thoracic Society; MR= Multiresistant; MRSA= Methicilin-resistant *Staphylococcus aureus.*

**S7 Table - Microbiological data according to survival of critically ill cancer patients admitted in the ICU with pneumonia with microbiological confirmation**

|  | **All Patients with microbiological confirmation**  **n= 169**  **(100%)** | **Survivors**  **n= 53**  **(31.4%)** | **Nonsurvivors**  **n= 116**  **(68.6%)** | **P Value*** |
| --- | --- | --- | --- | --- |
| **Adequate antibiotic therapy^1^** | 132  (78.1%) | 41  (77.4%) | 91  (78.4%) | 0.999 |
| **Positive blood culture** | 40  (23.7%) | 15  (28.3%) | 25  (21.6%) | 0.338 |
| **Gram negative** | 97  (57.4%) | 34  (64.2%) | 63  (54.3%) | 0.245 |
| ***Pseudomonas aeruginosa*** | 41  (24.3%) | 10  (18.9%) | 31  (26.7%) | 0.335 |
| ***Klebsiella pneumoniae*** | 15  (8.9%) | 4  (7.5%) | 11  (9.5%) | 0.779 |
| **Gram positive** | 69  (40.8%) | 22  (41.5%) | 47  (40.5%) | 0.999 |
| ***Staphylococcus aureus*** | 42  (24.9%) | 12  (22.6%) | 30  (25.9%) | 0.705 |
| ***Streptococcus pneumoniae*** | 21  (12.4%) | 10  (18.9%) | 11  (9.5%) | 0.129 |
| **MR Pathogens^2^** | 23  (13.6%) | 6  (11.3%) | 17  (14.7%) | 0.636 |
| **MRSA** | 11  (6.5%) | 3  (5.7%) | 8  (6.9%) | 0.999 |
| **ATS Guideline adherence^3^** | 26  (15.4%) | 11  (20.8%) | 15  (12.9%) | 0.250 |
| **Macrolide use** | 28  (16.6%) | 10  (18.9%) | 18  (15.5%) | 0.657 |
| **Atypical pathogen coverage** | 60  (35.5%) | 22  (41.5%) | 38  (32.8%) | 0.301 |
| **Only quinolone use** | 18  (10.7%) | 6  (11.3%) | 12  (10.3%) | 0.999 |
| **Number of antimicrobial drugs** |  |  |  |  |
| **1** | 87  (51.5%) | 25  (47.2%) | 62  (53.4%) | 0.508 |
| **2** | 61  (36.1%) | 20  (37.7%) | 41  (35.3%) |  |
| **> 2** | 21  (12.4%) | 8  (15.1%) | 13  (11.2%) |  |

*1- Adequate empiric antibiotic treatment was based in the sensitivity test of the identified bacteria.*

*2- The MR pathogens were defined as non-susceptibility to at least one agent in three or more antimicrobial categories.* ^22^

*3- ATS/IDSA guidelines adherence was based in definitions of empiric antimicrobial treatment for CAP and HCAP.* ^5^*,* ^20^

Definition of abbreviations: ATS= American Thoracic Society; MR= Multiresistant; MRSA= Methicilin-resistant *Staphylococcus aureus.*

**S8 Table - Demographic and clinical variables of patients admitted in the ICU with pneumonia with microbiological confirmation**

|  | **All Patients with microbiological confirmation**  **n=169**  **(100%)** | **Survivors**  **n=53**  **(31%)** | **Nonsurvivors**  **n= 116**  **(69%)** | **P Value*** |
| --- | --- | --- | --- | --- |
| **Age (years)** | 67  (58 – 73) | 67  (49 – 72.5) | 67.5  (59 – 74.75) | 0.299 |
| **Male gender** | 105  (62%) | 34  (64%) | 71  (61%) | 0.736 |
| ***Performance Status**** |  |  |  |  |
| **0-1** | 83  (49%) | 33  (62%) | 50  (43%) | 0.031 |
| **2-4** | 84  (50%) | 19  (36%) | 65  (56%) |  |
| **Previous Hospitalization^1^** | 57  (34%) | 17  (32%) | 40  (35%) | 0.861 |
| **Previous Chemotherapy^2^** | 49  (29%) | 10  (19%) | 39  (34%) | 0.067 |
| **PreviousRadiation Therapy^3^** | 17  (10%) | 6  (11%) | 11  (10%) | 0.784 |
| **Attended a hospital, nursing home or hemodialysis clinic** | 18  (11%) | 9  (17%) | 9  (8%) | 0.104 |
| **Solid tumors** | 119  (70%) | 34  (64%) | 85  (73%) | 0.276 |
| **Hematological malignancies** | 52  (31%) | 20  (38%) | 32  (28%) |  |
| **LOS prior ICU** | 1  (0 – 2) | 1  (0 – 2.5) | 1  (0 – 2) | 0.282 |
| **Charlson comorbidity index** | 3  (2 – 5) | 3  (2 – 4) | 3  (2 – 6) | 0.289 |
| **Neutropenia** | 16  (10%) | 6  (11%) | 10  (9%) | 0.580 |
| **Septic shock at ICU admission** | 140  (83%) | 37  (70%) | 103  (89%) | 0.004 |
| **SOFA D1 – points** | 8  (5 – 11) | 6  (4.5 – 9.5) | 8  (6 – 11.75) | 0.003 |
| **SAPS II – points** | 51  (42 – 61) | 48  (37 – 54) | 52  (45.75 – 65) | <0.001 |
| **Ventilatory support category** |  |  |  |  |
| **None** | 6  (4%) | 5  (9%) | 1  (1%) | 0.012 |
| **Exclusive NIV** | 13  (8%) | 10  (19%) | 3  (3%) | <0.001 |
| **NIV followed by MV** | 27  (16%) | 9  (17%) | 18  (16%) | 0.823 |
| **Invasive Mechanical Ventilation** | 150  (89%) | 38  (71.7%) | 112  (96.6%) | <0.001 |
| **RRT** | 55  (33%) | 8  (15%) | 47  (41%) | <0.001 |
| **Corticosteroids use 30 daysbefore** | 45  (27%) | 13  (25%) | 32  (28%) | 0.712 |

*1- Previous hospitalization is defined when a patient was hospitalized in an acute care hospital for two or more days within 90 days of the infection.*

*2- Previous chemotherapy is defined as chemotherapy within the past 30 days of the current infection.*

*3- Previous radiation therapy is defined as radiation therapy within the past 30 days of the current infection.*

Definition of abbreviations: LOS= length of stay; ICU= intensive care unit; NIV= noninvasive ventilation; SOFA score= sequential organ failure assessment score; SAPS score= simplified acute physiology score; RRT= renal replacement therapy

**S9 Table - Demographic and clinical variables of patients admitted in the ICU with pneumonia and classified according to inclusion period 2002-2005 and 2006-2013**

|  | **Inclusion period**  **2002-2005**  **n= 74**  **(23%)** | **Inclusion period**  **2006-2013**  **n= 251**  **(77%)** | **P Value*** |
| --- | --- | --- | --- |
| **Age (years)** | 64  (46.75 – 70) | 67  (57 – 75) | 0.015 |
| **Male gender** | 42  (57%) | 161  (64%) | 0.275 |
| ***Performance Status*** |  |  |  |
| **0-1** | 32  (43%) | 142  (57%) | 0.047 |
| **2-4** | 42  (57%) | 106  (42%) |  |
| **Solid tumors** | 54  (73%) | 177  (71%) | 0.771 |
| **Hematological malignancies** | 20  (27%) | 76  (30%) |  |
| **Hospital LOS prior ICU (days)** | 1  (0–2) | 1  (0–2) | 0.625 |
| **Charlson comorbidity** | 3  (2–4.25) | 3  (2–6) | 0.451 |
| **Neutropenia** | 11  (15%) | 24  (10%) | 0.204 |
| **Septic shock at ICU admission** | 68  (92%) | 176  (70%) | <0.001 |
| **SOFA D1 – points** | 7  (5–11) | 7  (5–10) | 0.333 |
| **SAPS II – points** | 51  (43–63) | 49  (37.5– 60) | 0.072 |
| **Ventilatory support category** |  |  |  |
| **None** | 3  (4%) | 20  (8%) | 0.311 |
| **Exclusive NIV** | 0  (0%) | 40  (16%) | <0.001 |
| **NIV followed by MV** | 3  (4%) | 40  (16%) | 0.006 |
| **MV** | 71  (96%) | 191  (76%) | <0.001 |
| **RRT** | 15  (20%) | 73  (29%) | 0.140 |
| **Corticosteroids use 30 days before** | 11  (15%) | 86  (34%) | 0.001 |
| **ICU mortality** | 35  (47%) | 114  (45%) | 0.792 |
| **Hospital mortality** | 52  (70%) | 159  (63%) | 0.332 |
| **ICU LOS (days)** | 9  (4–15) | 7  (3–15) | 0.370 |
| **Hospital LOS (days)** | 12  (6.75–30) | 16  (8–32) | 0.130 |

Definition of abbreviations: LOS= length of stay; ICU= intensive care unit; NIV= noninvasive ventilation; SOFA score D1= sequential organ failure assessment score in first day at ICU; SAPS II score= simplified acute physiology score; RRT= renal replacement therapy

**S10 Table - Microbiological data of patients admitted in the ICU with pneumonia and classified according to inclusion period 2002-2005 and 2006-2013**

|  | **Inclusion Period**  **2002 - 2005**  **n= 74**  **(23%)** | **Inclusion Period**  **2006 - 2013**  **n= 251**  **(77%)** | **P Value*** |
| --- | --- | --- | --- |
| **Adequate antibiotic therapy^1^** | 34  (87%) | 103  (79%) | 0.356 |
| **Positive blood culture** | 4  (10%) | 36  (28%) | 0.031 |
| **Gram negative** | 28  (72%) | 71  (54%) | 0.064 |
| ***Pseudomonas aeruginosa*** | 14  (36%) | 27  (21%) | 0.058 |
| ***Klebsiella pneumoniae*** | 4  (10%) | 11  (8%) | 0.750 |
| **Gram positive** | 18  (46%) | 51  (39%) | 0.460 |
| ***Staphylococcus aureus*** | 12  (31%) | 30  (23%) | 0.397 |
| ***Streptococcus pneumoniae*** | 6  (15%) | 15  (12%) | 0.580 |
| **MR Pathogens^2^** | 4  (10%) | 19  (15%) | 0.602 |
| **MRSA** | 3  (8%) | 8  (6%) | 0.716 |
| **ATS Guideline adherence^3^** | 5  (7%) | 3  (2%) | 0.017 |
| **Macrolide use** | 3  (4%) | 63  (25%) | <0.001 |
| **Atypical pathogen coverage** | 23  (31%) | 93  (37%) | 0.408 |
| **Only quinolone use** | 20  (27%) | 30  (12%) | 0.003 |
| **Number of antimicrobial drugs** |  |  |  |
| **1** | 31  (42%) | 126  (50%) | 0.235 |
| **2** | 35  (47%) | 89  (36%) |  |
| **> 2** | 8  (11%) | 37  (15%) |  |

*1- Adequate empiric antibiotic treatment was based in the sensitivity test of the identified bacteria.*

*2- The MR pathogens were defined as non-susceptibility to at least one agent in three or more antimicrobial categories. {Magiorakos:2012be}*

*3- ATS/IDSA guidelines adherence was based in definitions of empiric antimicrobial treatment for CAP and HCAP. {AmericanThoracicSociety:2005kw}, {Mandell:2007ik}*

Definition of abbreviations: ATS= American Thoracic Society; MR= Multiresistant; MRSA= Methicilin-resistant *Staphylococcus aureus.*
