# Supplementary material for: Clinical Outcomes and Microbiological Characteristics of Severe Pneumonia in Cancer Patients: A Prospective Cohort Study
Source: PLoS One. 2015 Mar 24;10(3):e0120544. doi: 10.1371/journal.pone.0120544 (PMC4372450; doi:10.1371/journal.pone.0120544)
Supplement: S2 Table — 1- Adequate empiric antibiotic treatment was based in the sensitivity test of the identified bacteria. 2- The MR pathogens were defined as non-susceptibility to at least one agent in three or more antimicrobial categories. {Magiorakos:2012be} 3- ATS/IDSA guidelines adherence was based in definitions of empiric antimicrobial treatment for CAP and HCAP. {AmericanThoracicSociety:2005kw}, {Mandell:2007ik}. Definition of abbreviations: ATS = American Thoracic Society; CAP = Community Acquired Pneumonia; HCAP = Healthcare-associated Pneumonia; MR = Multiresistant; MRSA = Methicilin-resistant Staphylococcus aureus. (DOCX) [file pone.0120544.s002.docx]

**S2 Table – Microbiological data according to the ATS/IDSA classification of Community-Acquired Pneumonia (CAP) and Healthcare-Associated Pneumonia (HCAP)**

|  | **CAP n= 132 (41%)** | **HCAP n= 193 (59%)** | **P Value*** |
| --- | --- | --- | --- |
| **Adequate antibiotic therapy^1^** | 58 (81%) | 78 (80%) | 0.999 |
| **Positive blood culture** | 16 (22%) | 24 (25%) | 0.855 |
| **Gram negative** | 50 (69%) | 49 (50%) | 0.012 |
| ***Pseudomonas aeruginosa*** | 19 (26%) | 22 (22%) | 0.589 |
| ***Klebsiella pneumoniae*** | 6 (8%) | 9 (9%) | 0.999 |
| **Gram positive** | 28 (39%) | 41 (42%) | 0.753 |
| ***Staphylococcus aureus*** | 15 (21%) | 27 (28%) | 0.370 |
| ***Streptococcus pneumoniae*** | 10 (14%) | 11 (11%) | 0.642 |
| **MR Pathogens^2^** | 9 (13%) | 14 (14%) | 0.823 |
| **MRSA** | 3 (4%) | 8 (8%) | 0.359 |
| **ATS Guidelines adherence^3^** | 50 (38%) | 3 (2%) | <0.001 |
| **Macrolide use** | 25 (19%) | 41 (21%) | 0.675 |
| **Atypical pathogen coverage** | 57 (43%) | 59 (31%) | 0.025 |
| **Only quinolone use** | 24 (18%) | 6 (3%) | <0.001 |
| **Number of antimicrobial agents** |  |  |  |
| **1** | 75 (57%) | 79 (41%) | 0.007 |
| **2** | 44 (33%) | 82 (43%) |  |
| **> 2** | 12 (9%) | 32 (17%) |  |

*1- Adequate empiric antibiotic treatment was based in the sensitivity test of the identified bacteria.*

*2- The MR pathogens were defined as non-susceptibility to at least one agent in three or more antimicrobial categories. {Magiorakos:2012be}*

*3- ATS/IDSA guidelines adherence was based in definitions of empiric antimicrobial treatment for CAP and HCAP. {AmericanThoracicSociety:2005kw}, {Mandell:2007ik}*

Definition of abbreviations: ATS= American Thoracic Society; CAP= Community Acquired Pneumonia; HCAP= Healthcare-associated Pneumonia; MR= Multiresistant; MRSA= Methicilin-resistant *Staphylococcus aureus.*
