# Supplementary material for: Clinical Outcomes and Microbiological Characteristics of Severe Pneumonia in Cancer Patients: A Prospective Cohort Study
Source: PLoS One. 2015 Mar 24;10(3):e0120544. doi: 10.1371/journal.pone.0120544 (PMC4372450; doi:10.1371/journal.pone.0120544)
Supplement: S3 Table — Definition of abbreviations: CAP = community-acquired pneumonia; LOS = length of stay; ICU = intensive care unit; NIV = noninvasive ventilation; SOFA score D1 = sequential organ failure assessment score in first day at ICU; SAPS II score = simplified acute physiology score; RRT = renal replacement therapy. (DOCX) [file pone.0120544.s003.docx]

**S3 Table – Demographic and clinical variables of Community-Acquired Pneumonia (CAP) patients and characteristics associated with hospital mortality**

|  | **All CAP Patients**  **n= 132 (100%)** | **Survivors n= 47 (36%)** | **Nonsurvivors**  **n= 85 (64%)** | **P Value*** |
| --- | --- | --- | --- | --- |
| **Age (years)** | 67 (58– 75) | 66 (58 – 74) | 68 (57.5 – 75) | 0.992 |
| **Male gender** | 94 (71%) | 33 (70%) | 61 (72%) | 0.844 |
| **Performance Status*** |  |  |  |  |
| **0-1** | 69 (52%) | 30 (64%) | 39 (46%) | 0.068 |
| **2-4** | 62 (47%) | 17 (36%) | 45 (53%) |  |
| **Solid tumors** | 100 (76%) | 36 (77%) | 64 (75%) | 0.999 |
| **Hematological malignancies** | 32 (24%) | 11 (23%) | 21 (25%) |  |
| **LOS prior ICU (days)** | 1 (0 – 2) | 1 (0 – 2) | 1 (0 – 2) | 0.621 |
| **Charlson comorbidity Index (points)** | 3 (2 – 4) | 3 (2 – 4) | 3 (2 – 4) | 0.278 |
| **Neutropenia** | 6 (5%) | 1 (2%) | 5 (6%) | 0.421 |
| **Septic shock at ICU admission** | 108 (82%) | 31 (66%) | 77 (91%) | <0.001 |
| **SOFA D1 (points)** | 7 (5 – 10) | 6 (4 – 9) | 8 (5 – 10.5) | 0.013 |
| **SAPS II (points)** | 49 (40 – 59) | 44 (35 – 53) | 52 (44 – 65) | <0.001 |
| **Ventilatory support category** |  |  |  |  |
| **None** | 4 (3%) | 3 (6%) | 1 (2%) | 0.129 |
| **NIV, only** | 13 (10%) | 11 (23%) | 2 (2%) | <0.001 |
| **NIV followed by MV** | 23 (17%) | 7 (15%) | 16 (19%) | 0.638 |
| **MV, only** | 115 (87%) | 33 (70%) | 82 (97%) | <0.001 |
| **RRT** | 37 (28%) | 5 (11%) | 32 (38%) | <0.001 |
| **Corticosteroids use 30 days before hospital admission** | 19 (14%) | 8 (17%) | 11 (13%) | 0.610 |

Definition of abbreviations: CAP= community-acquired pneumonia; LOS= length of stay; ICU= intensive care unit; NIV= noninvasive ventilation; SOFA score D1= sequential organ failure assessment score in first day at ICU; SAPS II score= simplified acute physiology score; RRT= renal replacement therapy
