# Supplementary material for: Clinical Outcomes and Microbiological Characteristics of Severe Pneumonia in Cancer Patients: A Prospective Cohort Study
Source: PLoS One. 2015 Mar 24;10(3):e0120544. doi: 10.1371/journal.pone.0120544 (PMC4372450; doi:10.1371/journal.pone.0120544)
Supplement: S4 Table — Definition of abbreviations: HCAP = healthcare-associated pneumonia; LOS = length of stay; ICU = intensive care unit; NIV = noninvasive ventilation; SOFA score D1 = sequential organ failure assessment score in first day at ICU; SAPS II score = simplified acute physiology score; RRT = renal replacement therapy. (DOCX) [file pone.0120544.s004.docx]

**S4 Table – Demographic and clinical variables of Healthcare-Associated Pneumonia (HCAP) patients and characteristics associated with hospital mortality**

|  | **All HCAP Patients**  **n= 193 (100%)** | **Survivors n= 67 (35%)** | **Nonsurvivors n= 126 (65%)** | **P Value*** |
| --- | --- | --- | --- | --- |
| **Age (years)** | 64 (54 – 73) | 63 (51 – 72) | 65 (57–74) | 0.158 |
| **Male gender** | 109 (57%) | 37 (55%) | 72 (57%) | 0.879 |
| **Performance Status*** |  |  |  |  |
| **0-1** | 105 (54%) | 38 (57%) | 67 (53%) | 0.652 |
| **2-4** | 86 (45%) | 28 (42%) | 58 (46%) |  |
| **Previous Hospitalization^1^** | 106 (55%) | 39 (58%) | 67 (53%) | 0.545 |
| **Previous Chemotherapy^2^** | 109 (57%) | 35 (52%) | 74 (59%) | 0.446 |
| **PreviousRadiation Therapy^3^** | 35 (18%) | 12 (18%) | 23 (18%) | 0.999 |
| **Attended a hospital, nursing home or hemodialysis clinic** | 49 (25%) | 26 (39%) | 23 (18%) | 0.003 |
| **Solidtumors** | 129 (67%) | 41 (61%) | 88 (70%) | 0.262 |
| **Hematological malignancies** | 64 (33%) | 26 (39%) | 38 (30%) |  |
| **LOS prior ICU (days)** | 1 (0–2) | 1 (0–2) | 1 (0–2) | 0.098 |
| **Charlson comorbidity Index (points)** | 3 (2–6) | 3 (2–6) | 3 (2–6) | 0.212 |
| **Neutropenia** | 29 (15%) | 10 (15%) | 19 (15%) | 0.999 |
| **Septic shock at ICU admission** | 136 (71%) | 32 (48%) | 104 (83%) | <0.001 |
| **SOFA D1 (points)** | 7 (5–10) | 6 (3 – 8) | 8 (6–11) | <0.001 |
| **SAPS II (points)** | 50 (38–61.75) | 45 (34 – 56) | 53 (42.5–67) | <0.001 |
| **Ventilatory support category** |  |  |  |  |
| **None** | 18 (9%) | 13 (19%) | 5 (4%) | <0.001 |
| **NIV, only** | 28 (15%) | 19 (28%) | 9 (7%) | <0.001 |
| **NIV followed by MV** | 27 (14%) | 8 (12%) | 19 (15%) | 0.665 |
| **MV, only** | 147 (76%) | 35 (52%) | 112 (89%) | <0.001 |
| **RRT** | 51 (26%) | 6 (9%) | 45 (36%) | <0.001 |
| **Corticosteroids use 30 days before hospital admission** | 78 (40%) | 26 (39%) | 52 (42%) | 0.760 |

Definition of abbreviations: HCAP= healthcare-associated pneumonia; LOS= length of stay; ICU= intensive care unit; NIV= noninvasive ventilation; SOFA score D1= sequential organ failure assessment score in first day at ICU; SAPS II score= simplified acute physiology score; RRT= renal replacement therapy
