# Supplementary material for: Clinical Outcomes and Microbiological Characteristics of Severe Pneumonia in Cancer Patients: A Prospective Cohort Study
Source: PLoS One. 2015 Mar 24;10(3):e0120544. doi: 10.1371/journal.pone.0120544 (PMC4372450; doi:10.1371/journal.pone.0120544)
Supplement: S5 Table — Definition of abbreviations: LOS = length of stay; ICU = intensive care unit; NIV = noninvasive ventilation; SOFA score D1 = sequential organ failure assessment score in first day at ICU; SAPS II score = simplified acute physiology score; RRT = renal replacement therapy. (DOCX) [file pone.0120544.s005.docx]

**S5 Table – Demographic and clinical variables of patients admitted in the ICU with pneumonia according to the type of cancer**

|  | **Solid tumors n= 229 (71%)** | **Hematological malignancies n= 96 (29%)** | **P Value*** |
| --- | --- | --- | --- |
| **Age (years)** | 66 (57.5–74) | 64.5 (45–72.75) | 0.030 |
| **Male gender** | 144 (63%) | 59 (62%) | 0.803 |
| ***Performance Status*** |  |  |  |
| **0-1** | 118 (52%) | 56 (58%) | 0.275 |
| **2-4** | 109 (48%) | 34 (35%) |  |
| **Hospital LOS prior ICU (days)** | 1 (0–2) | 1 (0–2) | 0.864 |
| **Charlson comorbidity** | 3 (2–6) | 3 (2–4) | <0.001 |
| **Neutropenia** | 12 (5%) | 23 (24%) | <0.001 |
| **Septic shock at ICU admission** | 168 (73%) | 76 (79%) | 0.326 |
| **SOFA D1 – points** | 7 (4–10) | 8 (6–11) | 0.006 |
| **SAPS II – points** | 49 (37–59) | 54 (45–66) | 0.002 |
| **Ventilatory support category** |  |  |  |
| **None** | 13 (6%) | 9 (9%) | 0.233 |
| **Exclusive NIV** | 29 (13%) | 12 (13%) | 0.999 |
| **NIV followed by MV** | 36 (16%) | 14 (15%) | 0.867 |
| **MV** | 187 (82%) | 75 (78%) | 0.539 |
| **RRT** | 53 (23%) | 35 (37%) | 0.020 |
| **Corticosteroids use 30 days before** | 53 (23%) | 44 (46%) | <0.001 |
| **ICU mortality** | 101 (44%) | 48 (50%) | 0.393 |
| **Hospital mortality** | 152 (66%) | 59 (62%) | 0.445 |
| **ICU LOS (days)** | 8 (3.5–15.5) | 9 (3–15.75) | 0.864 |
| **Hospital LOS (days)** | 14 (8–31) | 17 (8–34.75) | 0.709 |

Definition of abbreviations: LOS= length of stay; ICU= intensive care unit; NIV= noninvasive ventilation; SOFA score D1= sequential organ failure assessment score in first day at ICU; SAPS II score= simplified acute physiology score; RRT= renal replacement therapy
