# Supplementary material for: Clinical Outcomes and Microbiological Characteristics of Severe Pneumonia in Cancer Patients: A Prospective Cohort Study
Source: PLoS One. 2015 Mar 24;10(3):e0120544. doi: 10.1371/journal.pone.0120544 (PMC4372450; doi:10.1371/journal.pone.0120544)
Supplement: S6 Table — 1- Adequate empiric antibiotic treatment was based in the sensitivity test of the identified bacteria. 2- The MR pathogens were defined as non-susceptibility to at least one agent in three or more antimicrobial categories. {Magiorakos:2012be}. 3- ATS/IDSA guidelines adherence was based in definitions of empiric antimicrobial treatment for CAP and HCAP. {AmericanThoracicSociety:2005kw}, {Mandell:2007ik}. Definition of abbreviations: ATS = American Thoracic Society; MR = Multiresistant; MRSA = Methicilin-resistant Staphylococcus aureus. (DOCX) [file pone.0120544.s006.docx]

**S6 Table - Microbiological data of patients admitted in the ICU with pneumonia and classified according to the type of cancer**

|  | **Solid tumors n= 229 (71%)** | **Hematological**  **Malignancies n= 96 (29%)** | **P Value*** |
| --- | --- | --- | --- |
| **Adequate antibiotic therapy^1^** | 92 (79%) | 44 (85%) | 0.408 |
| **Positive blood culture** | 27 (23%) | 13 (25%) | 0.845 |
| **Gram negative** | 71 (61%) | 28 (54%) | 0.499 |
| ***Pseudomonas aeruginosa*** | 27 (23%) | 14 (27%) | 0.698 |
| ***Klebsiella pneumoniae*** | 10 (9%) | 5 (10%) | 0.778 |
| **Gram positive** | 46 (39%) | 23 (44%) | 0.612 |
| ***Staphylococcus aureus*** | 30 (26%) | 12 (23%) | 0.848 |
| ***Streptococcus pneumoniae*** | 13 (11%) | 8 (15%) | 0.455 |
| **MR Pathogens^2^** | 15 (13%) | 8 (15%) | 0.635 |
| **MRSA** | 7 (6%) | 4 (8%) | 0.739 |
| **ATS Guideline adherence^3^** | 39 (17%) | 14 (15%) | 0.626 |
| **Macrolide use** | 41 (18%) | 25 (26%) | 0.099 |
| **Atypical pathogen coverage** | 81 (35%) | 35 (37%) | 0.899 |
| **Only quinolone use** | 47 (21%) | 5 (5%) | <0.001 |
| **Number of antimicrobial drugs** |  |  |  |
| **1** | 115 (50%) | 39 (41%) | 0.144 |
| **2** | 89 (39%) | 37 (39%) |  |
| **> 2** | 24 (11%) | 20 (21%) |  |

*1- Adequate empiric antibiotic treatment was based in the sensitivity test of the identified bacteria.*

*2- The MR pathogens were defined as non-susceptibility to at least one agent in three or more antimicrobial categories.* {Magiorakos:2012be}

*3- ATS/IDSA guidelines adherence was based in definitions of empiric antimicrobial treatment for CAP and HCAP.* {AmericanThoracicSociety:2005kw}*,* {Mandell:2007ik}

Definition of abbreviations: ATS= American Thoracic Society; MR= Multiresistant; MRSA= Methicilin-resistant *Staphylococcus aureus.*
