# Supplementary material for: Clinical Outcomes and Microbiological Characteristics of Severe Pneumonia in Cancer Patients: A Prospective Cohort Study
Source: PLoS One. 2015 Mar 24;10(3):e0120544. doi: 10.1371/journal.pone.0120544 (PMC4372450; doi:10.1371/journal.pone.0120544)
Supplement: S7 Table — 1- Adequate empiric antibiotic treatment was based in the sensitivity test of the identified bacteria. 2- The MR pathogens were defined as non-susceptibility to at least one agent in three or more antimicrobial categories. 22 3- ATS/IDSA guidelines adherence was based in definitions of empiric antimicrobial treatment for CAP and HCAP. 5, 20 Definition of abbreviations: ATS = American Thoracic Society; MR = Multiresistant; MRSA = Methicilin-resistant Staphylococcus aureus. (DOCX) [file pone.0120544.s007.docx]

**S7 Table - Microbiological data according to survival of critically ill cancer patients admitted in the ICU with pneumonia with microbiological confirmation**

|  | **All Patients with microbiological confirmation n= 169 (100%)** | **Survivors n= 53 (31.4%)** | **Nonsurvivors n= 116 (68.6%)** | **P Value*** |
| --- | --- | --- | --- | --- |
| **Adequate antibiotic therapy^1^** | 132 (78.1%) | 41 (77.4%) | 91 (78.4%) | 0.999 |
| **Positive blood culture** | 40 (23.7%) | 15 (28.3%) | 25 (21.6%) | 0.338 |
| **Gram negative** | 97 (57.4%) | 34 (64.2%) | 63 (54.3%) | 0.245 |
| ***Pseudomonas aeruginosa*** | 41 (24.3%) | 10 (18.9%) | 31 (26.7%) | 0.335 |
| ***Klebsiella pneumoniae*** | 15 (8.9%) | 4 (7.5%) | 11 (9.5%) | 0.779 |
| **Gram positive** | 69 (40.8%) | 22 (41.5%) | 47 (40.5%) | 0.999 |
| ***Staphylococcus aureus*** | 42 (24.9%) | 12 (22.6%) | 30 (25.9%) | 0.705 |
| ***Streptococcus pneumoniae*** | 21 (12.4%) | 10 (18.9%) | 11 (9.5%) | 0.129 |
| **MR Pathogens^2^** | 23 (13.6%) | 6 (11.3%) | 17 (14.7%) | 0.636 |
| **MRSA** | 11 (6.5%) | 3 (5.7%) | 8 (6.9%) | 0.999 |
| **ATS Guideline adherence^3^** | 26 (15.4%) | 11 (20.8%) | 15 (12.9%) | 0.250 |
| **Macrolide use** | 28 (16.6%) | 10 (18.9%) | 18 (15.5%) | 0.657 |
| **Atypical pathogen coverage** | 60 (35.5%) | 22 (41.5%) | 38 (32.8%) | 0.301 |
| **Only quinolone use** | 18 (10.7%) | 6 (11.3%) | 12 (10.3%) | 0.999 |
| **Number of antimicrobial drugs** |  |  |  |  |
| **1** | 87 (51.5%) | 25 (47.2%) | 62 (53.4%) | 0.508 |
| **2** | 61 (36.1%) | 20 (37.7%) | 41 (35.3%) |  |
| **> 2** | 21 (12.4%) | 8 (15.1%) | 13 (11.2%) |  |

*1- Adequate empiric antibiotic treatment was based in the sensitivity test of the identified bacteria.*

*2- The MR pathogens were defined as non-susceptibility to at least one agent in three or more antimicrobial categories.* ^22^

*3- ATS/IDSA guidelines adherence was based in definitions of empiric antimicrobial treatment for CAP and HCAP.* ^5^*,* ^20^

Definition of abbreviations: ATS= American Thoracic Society; MR= Multiresistant; MRSA= Methicilin-resistant *Staphylococcus aureus.*
