# Supplementary material for: Clinical Outcomes and Microbiological Characteristics of Severe Pneumonia in Cancer Patients: A Prospective Cohort Study
Source: PLoS One. 2015 Mar 24;10(3):e0120544. doi: 10.1371/journal.pone.0120544 (PMC4372450; doi:10.1371/journal.pone.0120544)
Supplement: S8 Table — 1- Previous hospitalization is defined when a patient was hospitalized in an acute care hospital for two or more days within 90 days of the infection. 2- Previous chemotherapy is defined as chemotherapy within the past 30 days of the current infection. 3- Previous radiation therapy is defined as radiation therapy within the past 30 days of the current infection. Definition of abbreviations: LOS = length of stay; ICU = intensive care unit; NIV = noninvasive ventilation; SOFA score = sequential organ failure assessment score; SAPS score = simplified acute physiology score; RRT = renal replacement therapy (DOCX) [file pone.0120544.s008.docx]

**S8 Table - Demographic and clinical variables of patients admitted in the ICU with pneumonia with microbiological confirmation**

|  | **All Patients with microbiological confirmation**  **n=169 (100%)** | **Survivors n=53 (31%)** | **Nonsurvivors**  **n= 116 (69%)** | **P Value*** |
| --- | --- | --- | --- | --- |
| **Age (years)** | 67 (58 – 73) | 67 (49 – 72.5) | 67.5 (59 – 74.75) | 0.299 |
| **Male gender** | 105 (62%) | 34 (64%) | 71 (61%) | 0.736 |
| ***Performance Status**** |  |  |  |  |
| **0-1** | 83 (49%) | 33 (62%) | 50 (43%) | 0.031 |
| **2-4** | 84 (50%) | 19 (36%) | 65 (56%) |  |
| **Previous Hospitalization^1^** | 57 (34%) | 17 (32%) | 40 (35%) | 0.861 |
| **Previous Chemotherapy^2^** | 49 (29%) | 10 (19%) | 39 (34%) | 0.067 |
| **PreviousRadiation Therapy^3^** | 17 (10%) | 6 (11%) | 11 (10%) | 0.784 |
| **Attended a hospital, nursing home or hemodialysis clinic** | 18 (11%) | 9 (17%) | 9 (8%) | 0.104 |
| **Solid tumors** | 119 (70%) | 34 (64%) | 85 (73%) | 0.276 |
| **Hematological malignancies** | 52 (31%) | 20 (38%) | 32 (28%) |  |
| **LOS prior ICU** | 1 (0 – 2) | 1 (0 – 2.5) | 1 (0 – 2) | 0.282 |
| **Charlson comorbidity index** | 3 (2 – 5) | 3 (2 – 4) | 3 (2 – 6) | 0.289 |
| **Neutropenia** | 16 (10%) | 6 (11%) | 10 (9%) | 0.580 |
| **Septic shock at ICU admission** | 140 (83%) | 37 (70%) | 103 (89%) | 0.004 |
| **SOFA D1 – points** | 8 (5 – 11) | 6 (4.5 – 9.5) | 8 (6 – 11.75) | 0.003 |
| **SAPS II – points** | 51 (42 – 61) | 48 (37 – 54) | 52 (45.75 – 65) | <0.001 |
| **Ventilatory support category** |  |  |  |  |
| **None** | 6 (4%) | 5 (9%) | 1 (1%) | 0.012 |
| **Exclusive NIV** | 13 (8%) | 10 (19%) | 3 (3%) | <0.001 |
| **NIV followed by MV** | 27 (16%) | 9 (17%) | 18 (16%) | 0.823 |
| **Invasive Mechanical Ventilation** | 150 (89%) | 38 (71.7%) | 112 (96.6%) | <0.001 |
| **RRT** | 55 (33%) | 8 (15%) | 47 (41%) | <0.001 |
| **Corticosteroids use 30 daysbefore** | 45 (27%) | 13 (25%) | 32 (28%) | 0.712 |

*1- Previous hospitalization is defined when a patient was hospitalized in an acute care hospital for two or more days within 90 days of the infection.*

*2- Previous chemotherapy is defined as chemotherapy within the past 30 days of the current infection.*

*3- Previous radiation therapy is defined as radiation therapy within the past 30 days of the current infection.*

Definition of abbreviations: LOS= length of stay; ICU= intensive care unit; NIV= noninvasive ventilation; SOFA score= sequential organ failure assessment score; SAPS score= simplified acute physiology score; RRT= renal replacement therapy
