# Supplementary material for: Clinical Outcomes and Microbiological Characteristics of Severe Pneumonia in Cancer Patients: A Prospective Cohort Study
Source: PLoS One. 2015 Mar 24;10(3):e0120544. doi: 10.1371/journal.pone.0120544 (PMC4372450; doi:10.1371/journal.pone.0120544)
Supplement: S9 Table — Definition of abbreviations: LOS = length of stay; ICU = intensive care unit; NIV = noninvasive ventilation; SOFA score D1 = sequential organ failure assessment score in first day at ICU; SAPS II score = simplified acute physiology score; RRT = renal replacement therapy. (DOCX) [file pone.0120544.s009.docx]

**S9 Table - Demographic and clinical variables of patients admitted in the ICU with pneumonia and classified according to inclusion period 2002-2005 and 2006-2013**

|  | **Inclusion period**  **2002-2005 n= 74 (23%)** | **Inclusion period**  **2006-2013 n= 251 (77%)** | **P Value*** |
| --- | --- | --- | --- |
| **Age (years)** | 64 (46.75 – 70) | 67(57 – 75) | 0.015 |
| **Male gender** | 42 (57%) | 161 (64%) | 0.275 |
| ***Performance Status*** |  |  |  |
| **0-1** | 32 (43%) | 142 (57%) | 0.047 |
| **2-4** | 42 (57%) | 106 (42%) |  |
| **Solid tumors** | 54 (73%) | 177 (71%) | 0.771 |
| **Hematological malignancies** | 20 (27%) | 76 (30%) |  |
| **Hospital LOS prior ICU (days)** | 1 (0–2) | 1 (0–2) | 0.625 |
| **Charlson comorbidity** | 3 (2–4.25) | 3 (2–6) | 0.451 |
| **Neutropenia** | 11 (15%) | 24 (10%) | 0.204 |
| **Septic shock at ICU admission** | 68 (92%) | 176 (70%) | <0.001 |
| **SOFA D1 – points** | 7 (5–11) | 7 (5–10) | 0.333 |
| **SAPS II – points** | 51 (43–63) | 49 (37.5– 60) | 0.072 |
| **Ventilatory support category** |  |  |  |
| **None** | 3 (4%) | 20 (8%) | 0.311 |
| **Exclusive NIV** | 0 (0%) | 40 (16%) | <0.001 |
| **NIV followed by MV** | 3 (4%) | 40 (16%) | 0.006 |
| **MV** | 71 (96%) | 191 (76%) | <0.001 |
| **RRT** | 15 (20%) | 73 (29%) | 0.140 |
| **Corticosteroids use 30 days before** | 11 (15%) | 86 (34%) | 0.001 |
| **ICU mortality** | 35 (47%) | 114 (45%) | 0.792 |
| **Hospital mortality** | 52 (70%) | 159 (63%) | 0.332 |
| **ICU LOS (days)** | 9 (4–15) | 7 (3–15) | 0.370 |
| **Hospital LOS (days)** | 12 (6.75–30) | 16 (8–32) | 0.130 |

Definition of abbreviations: LOS= length of stay; ICU= intensive care unit; NIV= noninvasive ventilation; SOFA score D1= sequential organ failure assessment score in first day at ICU; SAPS II score= simplified acute physiology score; RRT= renal replacement therapy
