# Supplementary material for: Clinical Outcomes and Microbiological Characteristics of Severe Pneumonia in Cancer Patients: A Prospective Cohort Study
Source: PLoS One. 2015 Mar 24;10(3):e0120544. doi: 10.1371/journal.pone.0120544 (PMC4372450; doi:10.1371/journal.pone.0120544)
Supplement: S10 Table — 1- Adequate empiric antibiotic treatment was based in the sensitivity test of the identified bacteria. 2- The MR pathogens were defined as non-susceptibility to at least one agent in three or more antimicrobial categories. {Magiorakos:2012be}. 3- ATS/IDSA guidelines adherence was based in definitions of empiric antimicrobial treatment for CAP and HCAP. {AmericanThoracicSociety:2005kw}, {Mandell:2007ik}. Definition of abbreviations: ATS = American Thoracic Society; MR = Multiresistant; MRSA = Methicilin-resistant Staphylococcus aureus. (DOCX) [file pone.0120544.s010.docx]

**S10 Table - Microbiological data of patients admitted in the ICU with pneumonia and classified according to inclusion period 2002-2005 and 2006-2013**

|  | **Inclusion Period**  **2002 – 2005 n= 74 (23%)** | **Inclusion Period**  **2006 – 2013 n= 251 (77%)** | **P Value*** |
| --- | --- | --- | --- |
| **Adequate antibiotic therapy^1^** | 34 (87%) | 103 (79%) | 0.356 |
| **Positive blood culture** | 4 (10%) | 36 (28%) | 0.031 |
| **Gram negative** | 28 (72%) | 71 (54%) | 0.064 |
| ***Pseudomonas aeruginosa*** | 14 (36%) | 27 (21%) | 0.058 |
| ***Klebsiella pneumoniae*** | 4 (10%) | 11 (8%) | 0.750 |
| **Gram positive** | 18 (46%) | 51 (39%) | 0.460 |
| ***Staphylococcus aureus*** | 12 (31%) | 30 (23%) | 0.397 |
| ***Streptococcus pneumoniae*** | 6 (15%) | 15 (12%) | 0.580 |
| **MR Pathogens^2^** | 4 (10%) | 19 (15%) | 0.602 |
| **MRSA** | 3 (8%) | 8 (6%) | 0.716 |
| **ATS Guideline adherence^3^** | 5 (7%) | 3 (2%) | 0.017 |
| **Macrolide use** | 3 (4%) | 63 (25%) | <0.001 |
| **Atypical pathogen coverage** | 23 (31%) | 93 (37%) | 0.408 |
| **Only quinolone use** | 20 (27%) | 30 (12%) | 0.003 |
| **Number of antimicrobial drugs** |  |  |  |
| **1** | 31 (42%) | 126 (50%) | 0.235 |
| **2** | 35 (47%) | 89 (36%) |  |
| **> 2** | 8 (11%) | 37 (15%) |  |

*1- Adequate empiric antibiotic treatment was based in the sensitivity test of the identified bacteria.*

*2- The MR pathogens were defined as non-susceptibility to at least one agent in three or more antimicrobial categories. {Magiorakos:2012be}*

*3- ATS/IDSA guidelines adherence was based in definitions of empiric antimicrobial treatment for CAP and HCAP. {AmericanThoracicSociety:2005kw}, {Mandell:2007ik}*

Definition of abbreviations: ATS= American Thoracic Society; MR= Multiresistant; MRSA= Methicilin-resistant *Staphylococcus aureus.*
